# Methodology of the Sixth German Oral Health Study (DMS 6) to survey tooth and jaw misalignment

**DOI:** 10.1007/s00056-022-00436-0

**Published:** 2023-02-01

**Authors:** Andreas Rainer Jordan, Kathrin Kuhr, Cristiana Ohm, Nicolas Frenzel Baudisch

**Affiliations:** Institute of German Dentists, Universitätsstr. 73, 50931 Cologne, Germany

**Keywords:** Index of Complexity Outcome and Need, Epidemiology, Orthodontics, KIG classification, Health services research, Index of Complexity Outcome and Need, Epidemiologie, Kieferorthopädie, KIG-Klassifikation, Versorgungsforschung

## Abstract

**Purpose:**

The aim of this study was (1) to complete and update the oral-epidemiological data situation in Germany (descriptive epidemiology) and (2) to determine the need for orthodontic treatment provision based on the epidemiological data situation (health care epidemiology in the form of demand research).

**Methods:**

For this purpose, a longitudinal oral-epidemiological study and social science survey with a primary focus on tooth and jaw misalignment was conducted at a nationally representative level on 705 8‑ and 9‑year-old children across Germany.

**Results:**

The methodological principles of the oral-epidemiological study are described, with a focus on the calibration and reliability assessment results from the study dentists, sample weighting, a survey of nonrespondents to estimate the extent of the external validity of the study results, a description of the study participants, and realized cases, as well as information pertaining to the response rate and utilization.

**Conclusion:**

Based on the conducted analyses, it can be assumed that the examined 8‑ and 9‑year-old study participants are representative of the statistical population in Germany.

## Abstract (de)

**Zielsetzung:**

Ziele dieser Studie waren (1) die Vervollständigung und Aktualisierung der oral-epidemiologischen Datenlage in Deutschland (deskriptive Epidemiologie) und (2) die Ermittlung des kieferorthopädischen Versorgungsbedarfs auf Basis der epidemiologischen Datenlage (Versorgungsepidemiologie in Form von Bedarfsforschung).

**Methoden:**

Zu dieser Fragestellung wurden eine oral-epidemiologische Längsschnittstudie samt einer sozialwissenschaftlichen Befragung mit dem Schwerpunkt Zahn- und Kieferfehlstellungen auf bundesweit repräsentativer Ebene bei 705 8‑ und 9‑jährigen Kindern durchgeführt.

**Ergebnisse:**

Beschrieben werden die methodischen Grundlagen der oral-epidemiologischen Studie hinsichtlich der Ergebnisse der Kalibrierung und der Reliabilitätsanalyse der Studienzahnärzte, bezüglich der Stichprobengewichtung, der Nonrespondenten-Befragung zur Abschätzung des Ausmaßes der externen Validität der Studienergebnisse, hinsichtlich der Beschreibung der Studienteilnehmer und realisierten Fälle sowie der Angaben zur Rücklaufquote und zur Inanspruchnahme.

**Fazit:**

Aufgrund der durchgeführten Analysen kann davon ausgegangen werden, dass die untersuchten 8‑ und 9‑jährigen Studienteilnehmer repräsentativ für die statistische Grundgesamtheit in Deutschland sind.

## Introduction

Until now, tooth and jaw misalignment have only been investigated as part of the First German Oral Health Study in the states of the former Federal Republic of West Germany in 1989. There are no current population-wide data available on the prevalence of tooth and jaw misalignment in Germany. In particular, there are no systematic epidemiological data on tooth and jaw misalignment from the new federal states. Therefore, the overall orthodontic–epidemiological picture in Germany is incomplete—resulting in uncertainties in the planning of dental care provision. Furthermore, the composition of the general population following the reunification of Germany, due to the various waves of migration, is now subject to different dynamics. This further justifies the collection of new data, thus, providing us with the primary rationale for this study: to complete and update the oral-epidemiological data situation in Germany (descriptive epidemiology).

Alongside the scientific–epidemiological interest, various reports have raised the question of evidence-based practice in the field of orthodontics in recent years. In 2008, the Health Technology Assessment (HTA) of fixed orthodontic appliances by the German Institute for Medical Documentation and Information (DIMDI) concluded that “this reinforces the impression that there is a significant gap between the practical application of orthodontic measures and scientific research into their efficacy” [1]. Following an audit of the provision of orthodontic services, in its final report to the Federal Ministry of Health and the National Association of Statutory Health Insurance Funds, the Federal Audit Office warned of the lack of transparency in orthodontic care provision data [2].

A further report compiled for the Federal Ministry of Health (Bundesministerium für Gesundheit, BMG) by the Institute for Health and Social Research (IGES) on the benefits of orthodontic treatment measures proposed an array of measures to encourage the generation of more evidence and the inclusion of orthodontic topics in national epidemiological investigations [3]. This report raises the question of the actual need for care provision in Germany, from which we derive the second rationale for this study: Determining the need for orthodontic provision based on the oral–epidemiological data (health care epidemiology in the form of demand research).

## Methodology

Study planning was based on the methodology recommendation from the Epidemiology and Public Health Working Group at the German Society of Dentistry and Oral Medicine (Deutsche Gesellschaft für Zahn‑, Mund- und Kieferheilkunde, DGZMK) [4] and the Guidelines and Recommendations for ensuring Good Epidemiological Practice issued by the German Society for Epidemiology (Deutsche Gesellschaft für Epidemiologie, DGEpi) in 2018 [5]. The reporting follows the Statement Strengthening the Reporting of Observational Studies in Epidemiology (STROBE) [6].

### Study design

A longitudinal oral–epidemiological study and social science survey with a primary focus on tooth and jaw misalignment conducted at a nationally representative level.

#### Setting

The objective of the sampling model was to reflect the selected population group in Germany with as little distortion as possible. To implement the target objective, a two-stage sampling process via disproportionate stratified sampling was selected. In the first stage, a sample point was selected in each federal state and used to create area sampling (Table 1). Subsequently, in the second stage, a sample of persons was taken from the identified sample municipality. This was based on the register of persons from municipal registration authorities. This study aimed to encompass a net total of 670 study participants comprised of equal shares from the following age groups:

- Birth cohort 2012 (8 years old at the start of the field study in 2021)
- Birth cohort 2011 (9 years old at the start of the field study in 2021)

**Table 1:** Primary sampling units (municipalities) in the sample of the orthodontic module of the Sixth German Oral Health Study (DMS 6) with their respective federal state and simplified classification according to their population size (BIK categories) Studienzentren (Kommunen) in der Stichprobe für das kieferorthopädische Modul der Sechsten Deutschen Mundgesundheitsstudie (DMS 6) mit dem jeweiligen Bundesland und vereinfachter Klassifikation nach Bevölkerungsgröße (BIK-Kategorien)

Point	Municipality	Federal state	Simplified BIK categories
1	Reutlingen	Baden–Württemberg	Urban region
2	Nürnberg	Bavaria	Metropolitan region
3	Berlin	Berlin	Metropolitan region
4	Lübbenau/Spreewald	Brandenburg	Rural region
5	Bremen	Bremen	Metropolitan region
6	Hamburg	Hamburg	Metropolitan region
7	Ober-Ramstadt	Hesse	Urban region
8	Region Lubmin	Mecklenburg–Vorpommern	Rural region
9	Braunschweig	Lower Saxony	Urban region
10	Düsseldorf	North Rhine–Westphalia	Metropolitan region
11	Plaidt	Rhineland-Palatinate	Rural region
12	Saarbrücken	Saarland	Urban region
13	Hoyerswerda	Saxony	Rural region
14	Halle/Saale	Saxony–Anhalt	Urban region
15	Großhansdorf	Schleswig–Holstein	Metropolitan region
16	Altenburg	Thuringia	Rural region

To survey the 16 sample municipalities, two teams worked parallel to one another in the field, each consisting of a dentist, a person responsible for contacting participants, and an interviewer. Each team (i.e., dentist and interviewer) focused on one sample municipality per week across 6 working days. Following written and personal interviews, the study participants were asked to clean their teeth as thoroughly as possible to allow the dentist to assess their oral hygiene. The study participants were asked in advance to bring and use their own dental hygiene implements.

The subsequent dental medical examination was carried out in the following order:

- Orthodontic–clinical diagnosis,
- Orthodontic intraoral scan (for subsequent orthodontic model measurement),
- Caries and treatment,
- Plaque and gingival recession, and
- Bleeding of the gums.

The duration at the examination center, including registration, social science interview, and oral hygiene totaled about 45 min.

#### Study participants

The age of the study participants was selected to exclude, as far as possible, those already undergoing orthodontic treatment. This was done to ensure that treatment-naive tooth and jaw misalignment was recorded before any type of treatment had been administered; otherwise, this would result in systematic underestimation of severe disorders. For this reason, the age group of 8‑ to 9‑year-old children in Germany was selected as the statistical population for this study.

#### Inclusion and exclusion criteria

A target person must fulfill all the inclusion criteria listed below to be included in the study module:

- The target person is registered in one of the randomly selected sample municipalities.
- The target person was born in 2011 or 2012.
- The written consent form, signed by the target person’s parent or guardian, has been provided.

A target person was excluded from study participation if they fulfilled at least one of the following exclusion criteria:

- The target person, or their parent/guardian, have insufficient knowledge of the German language to participate in the study.
- Legal provisions.

#### Variables

The primary objective of this study was to collect data on the prevalence of tooth and jaw misalignment in 8‑ and 9‑year-old children in Germany (primary endpoint). For this purpose, the following indices were applied:

- KIG (Orthodontic Indication Groups) [7], and
- ICON (Index of Complexity Outcome and Need) [8].

The orthodontic indication groups were designated as the primary index for indicating prevalence within the context of the epidemiological question. The ICON index was used as a supporting index and primarily applied during international comparisons.

The secondary endpoint was to derive the need for orthodontic care provision from the prevalence of tooth and jaw misalignment data. The KIG and ICON indices were also used to answer this question.

#### Primary endpoint

The primary endpoint “Prevalence of Tooth and Jaw Misalignment” was operationalized as follows:

- KIG: KIG 1 vs. KIG 2 vs. KIG 3–5 (primary index).

In addition, the following was operationalized using scientifically broader criteria:

- ICON: Treatment complexity score easy, mild, moderate, difficult, very difficult (secondary index).

### Secondary endpoint

The secondary endpoint “Need for Orthodontic Treatment Provision” was based on statutory health care provider criteria and operationalized as follows:

- KIG 1–2 vs. KIG 3–5.

#### Bias

Parents who could not or chose not to participate in the study with their child were asked about the reasons for their nonparticipation and asked to answer a short questionnaire. The short questionnaire contained questions pertaining to their living situation, the parental assessment of the child’s dental condition, orthodontic treatment, frequency of dental visits, and their educational and professional background. This information made a comparison of nonrespondents and study participants possible using key indicators to provide insights into any systematic differences between the two groups.

#### Study size

The primary focus was the estimation of the prevalence of tooth and jaw misalignment using orthodontic indication group classification. In a clinical–epidemiological survey of 226 school children in classes 4 and 5 (9–13 years old) in 1993, 13.8% of the cases were classified as KIG 1, 34.6% as KIG 2, and 51.6% as KIG 3–5 (unpublished data from the National Association of Statutory Health Insurance Dentists). Further epidemiological surveys of primary school children identified percentages of KIG 1–2 classifications between 54% and 59%, and 41% to 46% for KIG 3–5 [9, 10]. To guarantee a reliable estimate, the standard error of prevalence should be no more than 10% of the prevalence. The standard error of prevalence to prevalence ratio is referred to as precision. To estimate the expected prevalence of 13% (KIG 1) with a confidence level of 95% and a standard error value of 1.3% (precision 10%), *n* = 670 study participants were necessary.

#### Quantitative variables

Orthodontic characteristics were surveyed in three different ways. The KIG and ICON endpoints were determined using the digital analytical model evaluation of the dental arches and occlusal interlocking in cases of habitual occlusion (Trios 3, 3Shape GmbH, Düsseldorf, Germany). Information on habits, dyskinesias, and dysfunctions was collected in interviews with study participants and dental medical diagnosis. During the dental medical diagnosis, cranial abnormalities, such as cleft lip and cleft palate, were also recorded. For ethical research reasons, comprehensive X‑ray examination was not possible within the scope of DMS 6. Generally, tooth retention, tooth displacement, hyperdontia and hypodontia, as listed in the system to classify the need for orthodontic treatment, recorded using the KIG classification system, can only be identified using radiological procedures. In cases where only a clinical examination is conducted, the prevalence is likely underestimated. For this reason, surveying of the mentioned findings did not take place. An exception that could be detected in the target age group without radiological diagnostics was ankylosis and partial retention of the six-year molars. The cephalometric analysis was conducted using calibrated and reliability-tested orthodontic specialists aided by OrthoAnalyzer analysis software (3Shape GmbH, Düsseldorf, Germany).

#### Statistical methods

To determine the need for orthodontic care provision as per KIG and ICON, and treatment complexity as per ICON, prevalences with the corresponding 95% confidence interval (CI) were reported. The results for the complete analysis set were stratified according to gender, region, and socioeconomic status. For the ICON total score, mean value with the corresponding confidence intervals, median and quartile, and minimum and maximum were given. In addition, the severity grade distribution for each individual KIG or ICON causal group was reported. To calculate the confidence interval for the prevalences, the one-sample case method from Newcombe and Altman was applied [11]. All reported *p*-values are two-sided. The analyses have an explorative character, and the *p*-values are only stated for descriptive purposes. The analyses were conducted using IBM SPSS Statistics for Windows, Version 26 (released 2019, IBM Corporation, Armonk, NY, USA), and R Version 3.5.3 (released 2019, R Core Team, R Foundation for Statistical Computing, Vienna, Austria).

## Results

### Calibration results

Reliability testing was carried out on 5 probands. All characteristics of interests were categorial; therefore, Cohen’s kappa (κ) was used for the analysis. Both the intrarater agreement and the interrater agreement of study dentists compared to the gold standard were of interest. The conventional Altman classification system was used for kappa value categorization [12, 13]:

- Kappa to 0.20: Poor agreement (*poor*)
- Kappa 0.21–0.40: Fair agreement (*fair*)
- Kappa 0.41–0.60: Moderate agreement (*moderate*)
- Kappa 0.61–0.80: Good agreement (*good*)
- Kappa >0.80: Very good agreement (*very good*)

The intrarater and interrater agreement in the sections tooth status and tooth-related findings were very good (tooth status: κ ≥ 0.92 and κ ≥ 0.93; tooth-related findings: κ ≥ 0.91 and κ ≥ 0.89). All study dentists were successfully assessed against the gold standard. In addition to the teams that carried out the field work, external orthodontic specialists were trained and calibrated to evaluate the intraoral scans. The analysis was carried out by the orthodontic specialists using OrthoAnalyzer analysis software (3Shape GmbH, Düsseldorf, Germany). Both intrarater and interrater comparisons of the evaluators against the gold standard were of interest. Intraclass correlation coefficients (ICC) were calculated as a statistical measure for the continuous characteristics (ICC type (3,1): two-way mixed, single measure). The Altman classification system, introduced in the previous segment, was used for ICC categorization [12, 13]. The calculated statistical measures for the assessment section in Table 2 are listed for all evaluators. To enable concomitant quality assessment of the surveyed data and to allow intervention for correction in the event of systematic deviation, 10% of all jaw models were subject to double measurement conducted by two different evaluators and no relevant deviations were detected.

**Table 2:** Orthodontic model analysis: results of the reliability analysis for the intra-individual perspective (within model analysts) and the inter-individual perspective (between model analysts) Kieferorthopädische Modellanalyse: Ergebnisse der Reliabilitätsanalyse für die intraindividuelle Perspektive (bei Modellauswertenden) und für die interindividuelle Perspektive (zwischen Modellauswertenden)

Section	Intra-Rater agreement	Inter-Rater agreement
Tooth width	Very good (ICC >0.99)	Very good (ICC >0.99)
Overjet	Very good (ICC >0.94)	Very good (ICC >0.84)
Overbite	Very good (ICC >0.96)	Very good (ICC >0.91)
High dental crowns	Very good (ICC >0.97)	Very good (ICC >0.94)
Front tooth segment	Very good (ICC >0.99)	Very good (ICC >0.99)
Support zone	Very good (ICC >0.97)	Very good (ICC >0.97)
Arch length 6‑year molars	Good to very good (ICC: 0.77–0.97)	Moderate to very good (ICC: 0.42–0.91)

*ICC* intraclass correlation coefficient

### Sample weighting

A weighting factor was used for all calculations to correct deviation between the analysis set and the population structure to provide representative statements for the group of 8‑ to 9‑year-old children in Germany. The calculation of the weighting factor was conducted in 3 stages. In the first stage, the sample design was taken into consideration. The sample design for DMS 6 was disproportionately applied to the federal states so that design weighting was calculated for four regions (northern Germany: Bremen, Hamburg, Mecklenburg–Vorpommern, Lower Saxony, Schleswig–Holstein; eastern Germany: Berlin, Brandenburg, Saxony, Saxony–Anhalt, Thuringia; southern Germany: Baden–Württemberg, Bavaria; western Germany: Hesse, North Rhine–Westphalia, Rhineland–Palatinate, Saarland).

Design weighting was inversely proportional to study participant selection probability. In the second stage, nonresponse weighting was applied. The aim was to align the net sample (study participants) with the (originally collected) gross sample. For this purpose, gross sample information and responses from the interviews with nonrespondents were used. To calculate the weighting, a multivariable logical regression model was adjusted to estimate the probability of study participation taking into account the explanatory variables of federal state, age, gender, and nationality. In the third stage, adjustment weighting was carried out. As orientation, information relating to the population data was drawn from official statistics. The characteristics of age, gender, region, nationality, education level of the father, and household size were taken into account. Final weighting was determined by multiplying the three weighting values and final standardization so that the weighting total corresponds to the extent of the analysis set (*n* = 705).

### Survey of nonrespondents

A survey was carried out to gain insights into the systematic differences between study participants and nonrespondents. The questionnaire focused on sociodemographic and oral health-related parameters. A total of 800 households were written to, and 165 parents/guardians returned the completed questionnaire. This corresponds to a nonrespondents’ survey response rate of 20.6%. As seen in Table 3, living situation distribution is similar in both groups. Only the percentage of children who live with their natural parents is 6 percentage points lower for study participants than children of parents/guardians who participated in the nonrespondents’ survey. Table 4 shows parental estimation of their child’s oral health. Table 5 shows the frequency of dental visits. This relates to complaint and control-orientated use of dental services. In this context, only minimal differences were observed between the two groups.

**Table 3:** Living situation of the study participants compared to that of nonrespondents Wohnsituation der Studienteilnehmer im Vergleich zu der der Nonrespondenten

With whom does your child primarily live?	Nonrespondent	Study participant
Natural parents	136 (82.4%)	548 (76.8%)
Mother and partner	7 (4.2%)	48 (6.7%)
Father and partner	–	1 (0.1%)
Mother	18 (10.9%)	90 (12.6%)
Father	–	5 (0.7%)
Grandparents/Other relatives	1 (0.6%)	–
Foster parents/Adoptive parents	–	3 (0.4%)
In a children’s home	1 (0.6%)	–
Information missing	2 (1.2%)	19 (2.7%)
Total	165 (100%)	714 (100%)

Stated as *n* (%)

**Table 4:** Estimation of the oral health status of the study participants by the parents/guardians compared to the nonrespondents Einschätzung des Mundgesundheitsstatus der Studienteilnehmenden durch die Eltern/Sorgeberechtigten im Vergleich zu den Nonrespondenten

How would you describe the conditions of your child’s teeth and gums?	Nonrespondent	Study participant
Very bad	–	3 (0.4%)
Bad	2 (1.2%)	13 (1.8%)
Moderate	17 (10.3%)	115 (16.1%)
Good	72 (43.6%)	405 (56.7%)
Very good	74 (44.8%)	174 (24.4%)
Information missing	–	4 (0.6%)
Total	165 (100%)	714 (100%)

**Table 5:** Frequency of dental visits by study participants compared to nonrespondents Häufigkeit der zahnärztlichen Untersuchungen von Studienteilnehmenden im Vergleich zu Nonrespondenten

Speaking in general: How would you complete the following sentence? I take my child to the dentist …	Nonrespondent	Study participant
I have never taken my child to the dentist	–	15 (2.1%)
… only when my child has problems with their teeth	5 (3.0%)	44 (6.2%)
… for occasional check-ups	17 (10.3%)	69 (9.7%)
… for regular check-ups	141 (85.5%)	586 (82.1%)
Information missing	2 (1.2%)	–
Total	165 (100%)	714 (100%)

### Study participants and realized cases

**Fig. 1:**
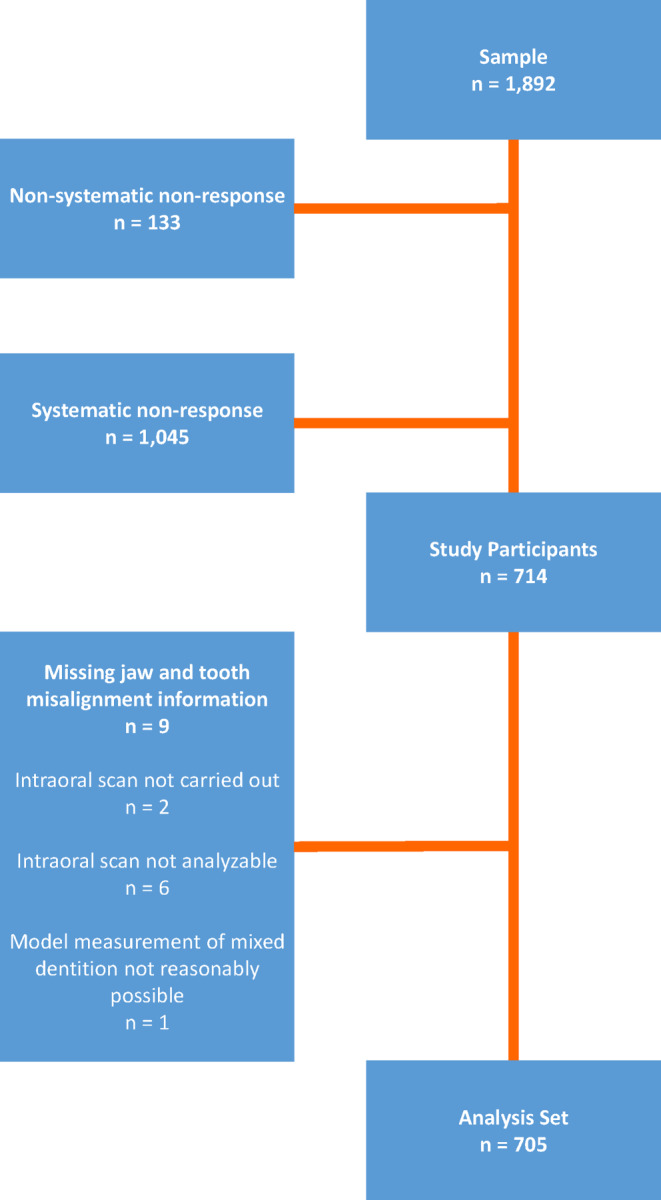
Flow chart. From the gross sample to analysis set of the German Oral Health Study (DMS) 6 orthodontic module Ablaufdiagramm. Von der Rohstichprobe zum Analyseset des kieferorthopädischen Moduls der Deutschen Mundgesundheitsstudie (DMS) 6

As can be seen in Fig. 1, a total of 1892 people were written to and invited to participate in the study. This case number corresponds to the unadjusted gross sample. In all, 133 study subjects were excluded from the unadjusted sample and classified as quality-neutral dropouts (QND). Fulfillment of the following criteria resulted in exclusion:

- Letter undeliverable,
- Deceased,
- Moved, no longer lives in the household,
- Poor command of the German language,
- In quarantine at the relevant time,
- Unable due to acute illness,
- Unable due to being in hospital,
- Unable due to undergoing a course of restorative treatment, or
- Unable due to chronic illness.

Alongside 133 quality-neutral dropouts, there were 1045 further exclusions–systematic dropouts. These included study subjects who could be classified using the following criteria:

- Address in original conditions,
- On vacation/travelling,
- Unable for other reasons,
- Not willing due to lack of time,
- Not willing due to being unconvinced of intent and purpose,
- Not willing for other reasons,
- No information on the target person, could not be contacted,
- Strictly rejected participation for data protection reasons,
- Strictly rejected participation for other reasons, complete objector,
- Did not appear at scheduled appointment, no information on the reasons why,
- Examination terminated, and
- Rejected participation because of doubts relating to the coronavirus disease 2019 (COVID-19) pandemic.

After QND and systematic dropout exclusion, 714 study participants remained.

For case definition, however, further differentiation was made between study participation and valid cases. Only study participant cases with the available jaw scan images were included in the statistical analysis. Using this definition, 714 study participants were registered and 705 cases were included in the data analysis.

### Response rate and utilization

The sample response rate reached 40.6% (Table 6). The response rate calculation was based both on response rate 2, in accordance with the American Association for Public Opinion Research [14] and the calculations stated in the cross-sectional survey in the study “Health of children and young people in Germany, 2nd wave” [15].

**Table 6:** Response rate calculation in accordance with the American Association for Public Opinion Research (AAPOR) Berechnung der Rücklaufquote in Anlehnung an die American Association for Public Opinion Research (AAPOR)

	Sample
Unadjusted gross sample	1892
Quality-neutral drop-outs	133
Adjusted gross sample	1759
Study participants	714
Nonparticipants	1045
Response rate	40.6%

## Discussion

In the first German Oral Health Study in 1989—as in this study—8- to 9‑year-old children underwent orthodontic examination. The most common finding was deep bite (34%), followed by enlarged overbite (17%), lateral crossbite (15%), and open bite (11%). According to Angle’s classification, 59% of children showed no neutral bite. Boys were significantly more likely to be diagnosed with a deep bite than girls. Habits, dyskinesias, and dysfunctions representing risk factors for tooth and jaw misalignment were very widespread: 53% of the children displayed dyskinesias such as lip and inner cheek biting; 44% of children exhibited fingernail biting; 19% of the 8‑ and 9‑year-old children reported occasionally sucking their thumb. Children who were identified with dysfunctions (orofacial dyskinesias) or, in particular, those who sucked their thumb, displayed significantly more tooth and jaw misalignments. At only 8 and 9 years old, 29% of those surveyed reported being unhappy with their tooth positioning. Dentition corresponding to the anatomical norm (eugnathic) was rare and fully observed in only 1% of children.

### Reliability testing

Except for 6‑year molar arch length, all examined characteristics displayed very good intrarater and interrater agreement. For the types of sagittal occlusive deviations (neutral/distal/mesial), the agreement was almost 100% with only one deviation observed across all evaluators and runs. Regarding the extent of sagittal occlusive deviations (neutral/less than cusp-on-cusp relation/cusp-on-cusp relation/more than cusp-on-cusp relation), in three of the 10 evaluated jaw halves, we observed interindividual and intraindividual deviations (cusp-on-cusp relation vs more than cusp-on-cusp relation). This is due to the fact that the digital models are difficult to judge objectively. When assessing whether contact point deviations of > 1 mm were evident, no intraindividual deviations were observed and interindividual deviations were observed in 3 of the 10 evaluated jaws.

### Survey of nonrespondents

While 90% of nonrespondents reported that their child’s tooth and gum status was very good, only 80% of study participants reported the same. A possible reason for this may be that the parents/guardians made more realistic oral hygiene statements due to the upcoming dental examination. Within the scope of the nonrespondents’ survey, distortion caused by a tendency to give socially desirable responses could also be a reason for the difference in estimation. However, overall, the analysis of the nonrespondents’ survey did not show systematic differences between study participants and the surveyed nonrespondents. Therefore, it can be assumed that there is no distortion of the study results stemming for the percentage of nonrespondents.

### Conclusions

Based on the conducted analysis, it can be assumed that the examined 8‑ and 9‑year-old children participating in the study are representative of the statistical population in Germany.
